# The value of qualitative research in randomized controlled trials involving drugs and medical devices: a systematic mapping review

**DOI:** 10.1017/S0266462326103936

**Published:** 2026-07-07

**Authors:** Yuan-Tao Huang, Benjamin Gregory, Sharon Greenwood, Evi Germeni

**Affiliations:** 1 Health Economics and Health Technology Assessment (HEHTA), School of Health and Wellbeing, University of Glasgow, Glasgow, UK; 2 Division of Psychological and Social Medicine, Leeds Institute of Health Sciences, School of Medicine, University of Leeds, Leeds, UK; 3 Public Health, School of Health and Wellbeing, University of Glasgow, Glasgow, UK

**Keywords:** qualitative research, randomized controlled trials, systematic review, drugs, medical devices

## Abstract

**Objective:**

The use of qualitative research with randomized controlled trials (RCTs) has grown exponentially over the last decade, but little is known about how it is contributing to trial procedures and interventions. This review systematically mapped the value that qualitative research has had in RCTs of drugs and medical devices.

**Methods:**

We searched five electronic databases (MEDLINE, EMBASE, PsycINFO, CINAHL, and ASSIA) and the NIHR Journals Library to identify original, peer-reviewed qualitative research conducted in the context of specific RCTs (pilot or full). Drawing on studies published between January 2019 and December 2020, we identified and classified examples illustrating how qualitative research had contributed to the trial. This created a list of questions qualitative methods can address in RCTs.

**Results:**

Of 207 included studies, only 22 percent explicitly articulated their value. Most frequently reported contributions included: (a) explaining trial findings; (b) ensuring that interventions meet the needs of key stakeholders; and (c) making the trial feasible and viable (e.g., through improved trial design and conduct). A total of 776 examples demonstrating how qualitative methods had enhanced the trial were classified into twenty-seven subcategories. A list of sixty potential questions was developed to assist future research design.

**Conclusions:**

The value of qualitative research in trials is expanding. While qualitative methods have traditionally been associated with complex interventions, this review highlights their relevance to trials involving drugs and devices. Future trials could benefit from expanding the use of qualitative methods to deepen understanding of outcomes, strengthen measurement validity, and capture experiences of conditions.

## Introduction

Qualitative research is increasingly conducted with randomized controlled trials (RCTs) to provide insights into stakeholders’ perceptions and experiences, as well as contextual factors influencing the design and implementation of trials and interventions (1). In a recently published methodological guidance on patient-focused drug development, the US Food and Drug Administration (FDA) highlighted the important benefits of using qualitative research during the process of intervention development and evaluation, which included, among others, identifying clinical outcomes that are important to patients and explaining low trial recruitment rates (2). The increased attention that regulatory authorities are currently paying to these issues indicates that the criteria of efficient health technologies are moving away from solely focusing on evidence of effectiveness to considering the multiple stakeholders involved, their behaviors, and the complexity of the real world in which they live (3).

There have been extensive discussions about the potential uses of qualitative research with trials. These typically focus on how qualitative methods can contribute to the generation of evidence of effectiveness before, during, or after large-scale trials (4). A 2013 mapping review conducted by O’Cathain et al. (1) identified twenty-two subcategories capturing the various ways that qualitative research can be used in conjunction with RCTs, including issues related to the intervention, the trial, outcomes, measures, and target conditions (1). Their results yielded an empirical foundation for previous debates, corroborating the idea that qualitative research has, in practice, been used in very diverse ways. Yet, the extent to which the potential value of qualitative research is currently realized remains poorly understood, largely due to how the findings of RCTs and associated qualitative research are presented in published literature. It is often unclear what follow-up work has arisen as a result of the qualitative findings, making it difficult to assess whether qualitative research has indeed fulfilled its promise (1). Since the use of qualitative methods in the context of RCTs has increased substantially over the last decade, it is worthwhile revisiting whether and how research practices may have shifted. This study maps the changing landscape a decade after O’Cathain et al.’s framework (1), which was based on publications from 2008 to 2010 (3;5).

Although an increasing number of qualitative studies have been conducted with clinical trials, their use remains uneven across different types of interventions. According to a systematic review of major trial registries, only around 5 percent of drug trials and 5 percent of device trials incorporated qualitative components (6). Similarly, a mapping review focusing only on RCTs estimated that these two categories of qualitative studies accounted for 27 percent altogether (7). The limited use of qualitative methods in the context of drug and device trials might be attributed to the perception that these interventions are highly standardized and less influenced by contextual variation. Yet, the complexity of drugs and devices may take different forms. For example, patients’ perceptions and experiences of drugs or devices might influence their adherence to long-term use (8–10). Similarly, health professionals’ views toward a drug or device can shape patients’ treatment choices (11–13), create hesitations around trial recruitment (14), compromise protocol fidelity (15), or even affect post-market surveillance (16). This study sought to refine the understanding of when and where qualitative methods can be used in conjunction with RCTs involving drugs or devices, to assess the extent to which existing qualitative studies have fulfilled their intended contributions, and to empirically develop a set of guiding questions to assist the design of future qualitative research.

## Methods

A systematic mapping review was conducted to characterize the uses and functions of qualitative research when conducted with contemporary RCTs. A protocol was developed before the study commencement; yet, given the nature of the review, this was not registered or published. The PRISMA Extension for Scoping Reviews (PRISMA-ScR) was used to guide the reporting of this review (Supplementary Table S1). As the primary goal of a mapping review is to describe the nature and characteristics of existing evidence (17) rather than synthesize identified studies, a critical appraisal is typically not recommended (18;19).

### Search strategy and eligibility criteria

Using an updated version of the search strategy developed by O’Cathain et al. (1) (Supplementary Table S2), we systematically searched five major electronic databases (MEDLINE, EMBASE, CINAHL, PsycINFO, and ASSIA), as well as the National Institute for Health and Care Research (NIHR) Journals Library, to identify original qualitative research that: (a) was published in English between 1 January 2019 and 31 December 2020; and (b) was directly associated with a specific RCT (pilot or full) in the field of health (i.e., the publication had to include the trial name and/or explicitly state the existence of a trial, or function as a precursor assessing the feasibility of a subsequent RCT). For a study to be considered “qualitative,” it had to employ recognized methods of both qualitative data collection (e.g., in-depth interviews, focus groups, and participant observation) and qualitative data analysis (e.g., thematic analysis and interpretative phenomenological analysis), as well as to include participant quotes (or other raw data) to support authors’ claims. Mixed methods studies were also eligible for inclusion, provided that the qualitative component was discussed in sufficient detail. When a single study was reported across multiple publications, only the one judged to be the most comprehensive was included (e.g., an NIHR report was typically preferred over a journal article). This review chose not to include gray literature to minimize heterogeneity in methodological transparency and reporting completeness.

### Screening and study selection

**Figure 1.**
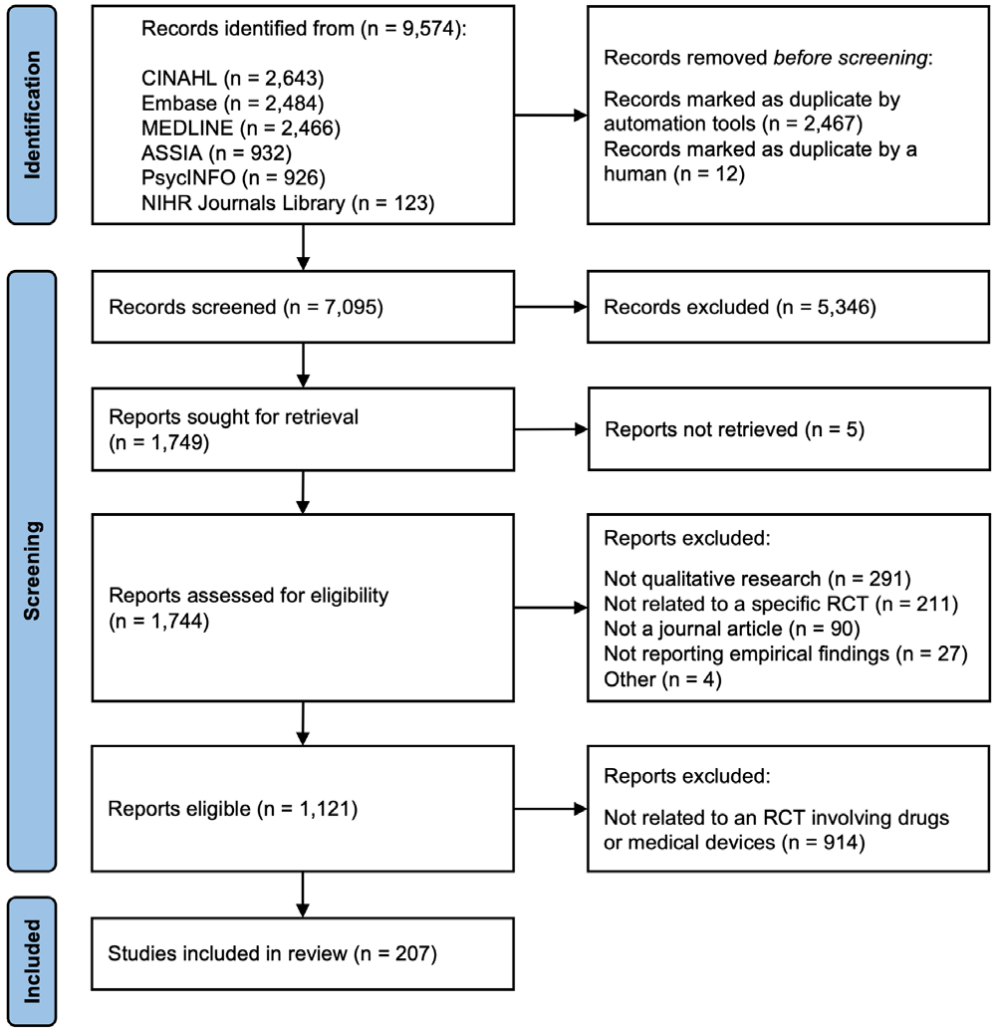
PRISMA flow diagram of study selection. Figure 1. long description.

A total of 9,574 citations identified from the electronic search were uploaded into Covidence, and duplicates were removed. The titles and abstracts of all 7,095 unique citations were screened by the first author (YTH), with a randomly selected 50 percent independently screened by a second reviewer (BG). To increase consistency in screening decisions, both reviewers initially screened twenty-five citations and discussed their rationale for including or excluding. Disagreements on study selection were resolved through consensus or discussion with a third reviewer (EG). The same process was followed when screening potentially relevant full-text articles, but reasons for exclusion were recorded at this stage. Studies were primarily excluded either because they were not qualitative (*n* = 291) or because they did not relate to a specific RCT (*n* = 211) (Figure 1).

Due to the large number of studies meeting our inclusion criteria (*n* = 1,121), we decided to focus exclusively on qualitative research conducted in the context of RCTs involving: (a) a drug (medicine or vaccine); (b) a conventional medical device; or (c) a digital device, as defined by the Medicines and Healthcare products Regulatory Agency (20). Post-hoc modifications to the inclusion and exclusion criteria are common in scoping/mapping reviews, given that study selection is not a strictly linear process (as is the case, for instance, with systematic reviews of effectiveness), but rather an iterative one (21). As the name suggests, mapping reviews seek to map the existing literature on a broad topic and, as the reviewers’ familiarity with the existing evidence landscape grows, decisions related to the scope of the review might be revisited. In our case, this involved restricting our scope to RCTs of drugs and medical devices, given that extensive qualitative research has already been conducted alongside RCTs of social and psychological interventions, whereas the implementation nuances and subjective experiences of using drugs and medical devices remain largely unexplored. A subset of 10 percent of eligible studies was double-checked by second reviewers (EG and SG), and there was full agreement on further exclusions. This left us with 207 papers for further review and analysis (20).

### Data extraction and analysis

We began by extracting the key characteristics of each study into an Excel spreadsheet. These included, for instance, information such as the journal of publication, data collection and analysis methods, timing of qualitative research (i.e., before, during, or after the trial), and therapeutic area of the intervention, according to the International Classification of Diseases (ICD-10) (22). The discussion and conclusion sections of all included studies were then scrutinized to understand the extent to which the potential value of using qualitative methods in the context of an RCT was empirically demonstrated. Similar to O’Cathain et al. (1), we did not focus on the value that the qualitative findings had in their own right, that is, the general contributions to knowledge that they made, but rather on instances where they had enhanced the research endeavor (e.g., by identifying ways to optimize trial recruitment and retention). As such, we also sought to discern what kind of follow-up actions (if any) were taken in response to the qualitative results.

Employing an approach similar to framework synthesis (23), we used O’Cathain et al.’s (1) framework as a “scaffold” for the initial data extraction. Each “example” of the contribution made by the qualitative research was derived through immersion in the included studies, particularly through repeated reading of their results and discussion sections. The representative cases, corresponding to the twenty-two subcategories identified by O’Cathain et al. (1), were read multiple times and served as “typical” cases for determining the appropriate subcategory for each example. Examples were categorized into the subcategory where the typical case and example were most similar. There were a few distinct examples that could not fit in the original framework; these were categorized into new subcategories (e.g., a new subcategory, called “private life influenced by the intervention,” was created for the example “glucose monitoring reduced conflict between pediatric diabetics and their parents” (24)). By merging existing subcategories and adding new ones, the original framework was iteratively adjusted and refined, resulting in a revised candidate framework, which covered a range of functions that qualitative research can serve in the context of contemporary trials. Twenty percent of the included studies were randomly selected and double-checked by a second reviewer (EG or SG), who read the full texts and independently extracted data, identified examples, and developed their own candidate subcategories. These new subcategories were then compared with the preliminary candidate subcategories developed by the first author. Disagreements were discussed internally within the research team, leading to the refinement of the category descriptions and final categorizations. This process aims to increase the consistency of the analysis and to ensure that the framework is not dependent on a single perspective. Thinking backwards, the example about glucose monitoring might address the question “what impact did the monitoring device have on parent–child relationship?”. This question, however, can be abstracted further to “what impact did the intervention have on interpersonal relationships and family life?”. We identified one such question for each example derived and compiled a list of issues that can be addressed by using qualitative research with clinical trials.

The entire research process was resource-demanding, lasting about 33 months from the initial testing of the search strategy to the completion of analysis and reporting.

## Results

### Characteristics of included studies

Of the 1,121 studies that met our inclusion criteria, 207 (18.5 percent) studies conducted in conjunction with RCTs involving drugs or medical devices were included in the final analysis. Among these, 91 were published in 2019 and 116 in 2020. The included studies were published across 120 academic journals. Sixty-five out of the 207 studies (31.4 percent) were conducted at the pretrial stage and were described as feasibility, pilot, or early-stage studies. However, based on the information reported in individual studies, most of the remaining 142 studies did not report start or end dates, making it difficult to determine whether the qualitative research was conducted during or after the trial. Identified qualitative studies were associated with 211 RCTs (as eight qualitative studies were related to more than one RCT, and eleven RCTs were each associated with multiple qualitative studies), and of which 191 trials (related to 189 qualitative studies) reported their registration year. Of these, 182 trials were registered between 2010 and 2020.

The 211 identified RCTs were conducted across fifty-nine countries around the world. However, the associated qualitative research was carried out in thirty-nine of these, with a significant number (*n* = 17) in countries classified as high-income economies according to the World Bank criteria (25). The countries where RCTs were conducted without a qualitative research component were mostly located in Central and Eastern Europe (*n* = 9), Asia (*n* = 3), and South America (*n* = 3). Although a total of thirty-seven qualitative studies were conducted in the context of large, international, multicenter trials, eighteen of these investigations were limited to a single country.

**Figure 2.**
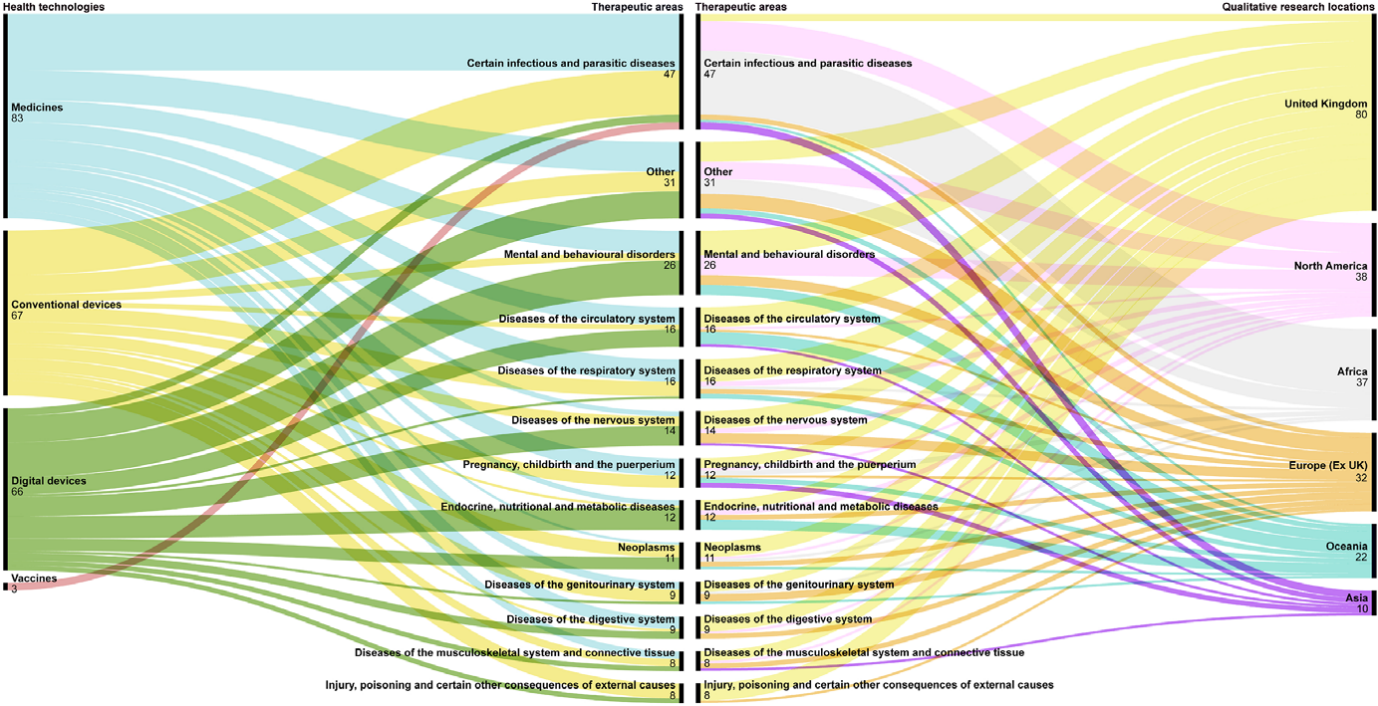
Distribution of qualitative studies across health technologies, therapeutic areas, and the locations of qualitative research. Figure 2. long description.

Figure 2 displays the distribution of qualitative studies across health technologies under study (i.e., medicines, vaccines, conventional medical devices, and digital devices), therapeutic areas that they targeted, and the locations of qualitative research. Eleven studies were counted more than once, as they included respondents from various locations. Wider lines represent a larger number of relevant studies. As shown on the left side of the diagram, studies were in relatively even distribution across RCTs of various health technologies (excluding vaccines). Notably, studies referring to digital devices (green lines) covered almost all therapeutic areas (except pregnancy and childbirth), although most of them primarily addressed psychiatric, circulatory, and neurological disorders. The lines on the right side of the diagram illustrate how qualitative studies in each region focused on similar therapeutic areas or differed from one another. For instance, infectious diseases were the focus of most studies undertaken in Africa, North America, and Asia. By comparison, studies conducted in the United Kingdom and Oceania were mostly related to mental health disorders and circulatory system diseases, respectively. Qualitative research with RCTs conducted in Europe demonstrated substantial breadth. Across all individually defined therapeutic areas, neurological and psychiatric disorders were the most frequently addressed. Although the aggregated “other” was the largest single line, it represents a consolidation of numerous conditions.

### The articulated value and potential contributions of qualitative research

Forty-six out of the 207 studies (22.2 percent) explicitly articulated the value of qualitative research to the specific trial and/or intervention by reporting follow-up actions that were implemented in response to the qualitative findings. Of these forty-six studies, twenty-seven were conducted at the pretrial stage. The values most frequently reported by researchers were: explaining trial findings (*n* = 22), including explanations of why the use of specific interventions is low (26); ensuring that interventions meet the needs of key stakeholders (*n* = 14), by suggesting improvements and identifying potential issues (27); and making a trial feasible and viable (*n* = 13), for example, through improved trial design and conduct based on the qualitative findings (28); Other values of the qualitative research, as mentioned by the original authors, included: increasing trial recruitment rates (*n* = 4) (29); improving participant informed consent (*n* = 4) (30); and making trials more sensitive and considerate toward people (*n* = 4) (e.g., by refining presentation strategies and improving communication experiences for both participants and recruiters (29)).

A total of 776 examples of the contribution made by employing qualitative research with RCTs were identified, then divided into twenty-seven subcategories according to their functions. Table 1 presents one representative example from each subcategory to illustrate a given function. Around two-thirds of the examples focused on the intervention content and delivery, whereas a very limited number of examples were classified into the categories of outcomes and measures of process and outcome. However, it is worth noting that the pretrial examples made a significant contribution to both categories. As precursors to full-scale RCTs, the pretrial examples were also frequently related to assessing the feasibility and acceptability of the trial in practice.

**Table 1.** The uses of qualitative research in the context of RCTs involving drugs and devices Table 1. long description.

Category	Subcategory	Description	Frequency in 776 examples (%)	Frequency in 243 pre-trial examples (%)	Example
Intervention content and delivery			493 (63.5%)	150 (61.7%)	
	Intervention development and adjustment^b^	The design, development, or adaptation of the intervention’s content or delivery	39 (5.0%)	14 (5.8%)	Kapoor et al. (31), as part of a pragmatic, double-blind RCT of controlling severe allergic asthma via a laminar airflow device, undertook interviews with 10 patients and 2 partners and 3 focus group discussions (FGDs) with 10 patients in total. The qualitative researchers identified the weight and mobility deficiencies of the intervention, which made cleaning difficult and prevented patients from using it in other rooms. They suggested improvements, such as extending the size of its shelf to function as a bedside table.
	Models, mechanisms, and underlying theory development^a^	Developing models, mechanisms of action, and underlying theories or concepts relating to an intervention in the context of a specific trial	25 (3.2%)	7 (2.9%)	Keene et al. (32), within a cluster RCT of increasing antiretroviral therapy compliance through patient clubs, applied 36 in-depth interviews with informants, health professionals, and participants involved in both arms. They illustrated that the trigger for patients in the 6-month refill to keep high treatment adherence is their misunderstanding about randomization, since they believed that the experimental arm was an accolade upgrade for patients who maintained good medication habits and those who failed to maintain a low viral load would lose their premium services.
	Perceived value and benefits of intervention^a^	Exploring stakeholders’ perceptions regarding the intervention’s benefits, including their extent, changes over time, etc.	109 (14.0%)	32 (13.2%)	Batchelor et al. (33), as part of a pragmatic RCT of ultraviolet B light therapy for vitiligo, conducted semi-structured interviews with 25 participants (or parents) and 10 commissioners, plus FGDs with 13 health professionals. They suggested that regular photographic documentation is an essential process for patients to become aware of (or validate) their perceived benefits of the intervention, particularly in the context of disruptive circumstances such as seasonal variations in skin color.
	Acceptability of intervention in principle^a^	Exploring stakeholder perceptions of the acceptability of an intervention in principle	5 (<1%)	4 (1.6%)	Laher et al. (34) undertook FGDs with 38 participants as part of a phase II/III RCT on an HIV vaccine to examine their acceptability of hypothetical interventions. They revealed that cultural beliefs regarding sexual openness and indiscretion make it unacceptable for patients to use administration that is visible to others (such as transdermal patches or vaginal gels). Additionally, long-acting injections were recommended since short-term preventive medication is less acceptable due to difficulty planning sexual activities.
	Feasibility and acceptability of intervention in practice^a^	Exploring stakeholder perceptions of the feasibility and acceptability of an intervention in practice	151 (19.5%)	50 (20.6%)	Kong et al. (35) undertook semi-structured interviews with 28 participants following an open-label RCT of screening skin cancer through mobile teledermoscopy. The qualitative research yielded insights into restrictions to the acceptability of the intervention, including the case of patients who live alone and may therefore have difficulty detecting skin lesions without the assistance of their partners or caregivers.
	Compliance with intervention^b^	Examining the fidelity, dose, and adherence of an intervention as implemented in a specific trial	43 (5.5%)	12 (4.9%)	Mupambireyi and Bernays (36), within an open-label RCT about children living with HIV, conducted qualitative longitudinal research through multiple methods, including audio diaries to examine the lived experience of using the interventions over time. The study identified several factors that may influence children’s treatment compliance over time. For example, these included instances where a caregiver was unable to fulfil caring responsibilities for a sibling following a change in personal circumstances. The findings also indicated that qualitative data collected in the absence of investigators may offer valuable insights into the full picture of compliance.
	Implementation of the intervention in the real world^a^	Identifying lessons for real-world implementation based on delivery of the intervention in the trial	64 (8.2%)	16 (6.6%)	Msimango et al. (37), as part of an open-label RCT testing viral load, undertook semi-structured interviews with 55 clients and 8 nurses plus FGDs for those clients. The qualitative research stated the reasons why large-scale implementation of point-of-care testing is challenging (e.g., the huge number of patients that need to be served and the limited human resources and quality assurance capacity available in South Africa).
	Perceptions and treatment choices between different interventions^c^	Identifying diverse perceptions and preferences for various interventions and stakeholder influences on treatment choices	43 (5.5%)	13 (5.3%)	Reeves et al. (38) undertook interviews with 37 patients from both arms and 18 clinical professionals from 3 sites as part of a pragmatic pilot RCT involving three wound dressing strategies. Although the objective was merely to ascertain the acceptability of study allocation, this qualitative research identified the comparative strengths of specific interventions. For instance, the ability to avoid changing and removing dressings was an advantage of “no dressing” and “glue” as compared to “simple dressing,” contributing to several benefits such as bathing quickly and without restriction.
	Private life influenced by the intervention^c^	Investigating the impact of the intervention on private space, interpersonal relationships, and family life	14 (1.8%)	2 (<1%)	Burckhardt et al. (39), as part of a cross-over RCT monitoring pediatric diabetes through remote equipment, used semi-structured interviews with 20 parents. They found that the intervention created a technically feasible environment for partners to balance their parenting responsibilities because it enabled both to monitor their children’s glucose levels using mobile devices at any time, although it could also result in a shift in the father’s role in care work from a nonparticipant to a supervisor.
Trial design, conduct, and processes			192 (24.7%)	63 (25.9%)	
	Recruitment and retention^a^	Describing barriers or solutions related to the recruitment and retention	48 (6.2%)	15 (6.2%)	Nikčević et al. (40) conducted semi-structured interviews with 27 pregnant women who agreed or declined to participate in a double-blind RCT preventing preeclampsia via low-dose aspirin. The qualitative research identified that health risks, extra services, and interventions with acceptable anxiety levels attracted people to join the study, whereas risks associated with pregnancy medication or using placebos, as well as satisfaction with the information provided during recruitment, impaired motivations to participate. Also, they observed that influential individuals, especially the partner, are key stakeholders in influencing participation decisions.
	Engagement and performance of health professionals in trial^c^	Exploring facilitators and barriers related to health professionals’ participation and performance in clinical trials	24 (3.1%)	7 (2.9%)	Dalton et al. (41), as part of a pragmatic open-label RCT assisting pharmacotherapy advice through software, undertook semi-structured interviews with 14 prescribers and 10 researchers from 6 sites. The qualitative research revealed important factors that influence whether prescribers use the allocated intervention in RCTs; additionally, they provided theoretical hypotheses for subsequent quantitative studies related to these factors and prescribers’ behaviors, as well as implementation recommendations for future trials.
	Preparation and training^c^	Identifying the impacts, challenges, needs, and suggestions of training and preparation on trials.	19 (2.4%)	5 (2.1%)	Pocock et al. (42), within a pragmatic double-blind RCT treating cardiac arrest patients out of hospital through adrenaline, used 4 FGDs with 44 participants in total. The qualitative study observed the benefits of using face-to-face training (reduced anxiety among ambulance staff about ethical dilemmas and improved trial performance) and provided insight into how to improve trial preparation.
	Feasibility and acceptability of the trial in principle^b^	Exploring stakeholders’ views of feasibility and acceptability of a trial design	5 (<1%)	5 (2.1%)	Alhowimel et al. (43), as part of a feasibility RCT on chronic low back pain, undertook semi-structured interviews with five patients and two doctors. The qualitative study identified multiple barriers to the successful implementation of a large-scale RCT in Saudi Arabia based on the feasibility trial.
	Feasibility and acceptability of the trial in practice^b^	Exploring stakeholders’ views of feasibility and acceptability of a trial design in practice	48 (6.2%)	19 (7.8%)	Pekmezaris et al. (44) undertook nine FGDs with various stakeholders during an 8-month iterative adaptation process of the intervention, plus FGDs and structured interviews with 12 patients in a pilot study that was preparing for an incoming RCT assisting Hispanic patients to manage type 2 diabetes through a digital application. The qualitative research indicated that the trial was acceptable overall, although some participants found both the consent process and survey time-consuming. Investigators modified the trial design based on qualitative findings, including reducing survey items and incorporating additional explanations about privacy protections, etc.
	Acceptability of post-research care arrangements^c^	Exploring stakeholders’ views on the acceptability of arrangements after the end of clinical studies	4 (<1%)	1 (<1%)	Odero et al. (45), within a phase III single-blind RCT treating HIV through antiretroviral therapy and primary care, conducted FGDs with 15 participants and in-depth interviews with 5 participants. They indicated that participants were very dissatisfied since the public centers did not offer the same high-quality health infrastructure, transportation arrangements, or frequent doctor–patient interaction as the trial centers did; thus, some trial participants refused to transit to public centers at the end of the trial, resulting in their deaths. The qualitative results yielded valuable insights into the factors influencing the acceptability of healthcare arrangements following the trial and the impact of acceptability on participants’ health.
	Ethical conduct^a^	Identifying moral concerns and ethical issues related to trial design and intervention delivery	20 (2.6%)	6 (2.5%)	Sherratt et al. (29), as part of a feasibility trial treating pediatric appendicitis, undertook semi-structured interviews with 35 health professionals plus parents and children from 28 families. They found that healthcare professionals failed to be transparent and balanced in providing information about the advantages and disadvantages of interventions in patient recruitment. The qualitative research also highlighted a divergence of views between children and guardians regarding treatment preferences and trial participation. However, children were often overlooked in discussions.
	Adaptation of trial conduct to local context^a^	Addressing local issues that may impact the feasibility of the trial	2 (<1%)	0	Dick et al. (46) conducted semi-structured interviews with 15 multidisciplinary members involved in the study, as part of a pragmatic stepped-wedge cluster RCT of a community case management smartphone application. They identified local factors that could affect assessment of the effectiveness of digital devices, including inadequate infrastructure, limited technological literacy, and perceptions of smartphones.
	Impact of trial on staff, researchers, or participants^a^	Understanding how the trial affects different stakeholders	17 (2.2%)	4 (1.6%)	Lewis et al. (47), as part of a pragmatic, open-label RCT on lower urinary tract symptoms, undertook semi-structured interviews with 50 patients who were invited to take part in the trial and 21 health professionals from 18 trial sites. They suggested that staff thought the trial delayed patients’ clinical pathways as some did not require a urodynamic test and required additional time for the extra work; however, for other doctors, it increased their use of the intervention in day-to-day practice after they saw its benefits.
	Role of trial components other than interventions^c^	Investigating the effects of nonintervention trial components	5 (<1%)	1 (<1%)	Katz et al. (48), within a phase III double-blind RCT on dapivirine vaginal rings, utilized 12 FGDs with 89 patients in total and in-depth interviews with 98 patients to understand the factors influencing compliance. They suggested that participants who knew each other previously often shared positive comments, while those who were unfamiliar shared negative comments during trial activities (such as tea parties and movie days). The qualitative research identified factors that may contribute to heterogeneity in treatment adherence level among enrolled patients, providing insight into the interpretation of trial results.
Outcomes			14 (1.8%)	8 (3.3%)	
	Breadth of outcomes^a^	Identifies the range of outcomes important to participants in the trial	8 (1.0%)	6 (2.5%)	Jones et al. (49), as part of a feasibility trial on juvenile idiopathic arthritis, used semi-structured interviews with participants from 15 families to determine which s were most significant to children and their parents. They suggested that rather than curing the disease, reducing the severity of the disease and flare recurrence were key outcomes for patients and their parents; and long-term sustained treatment effects (such as pain relief, relief from joint stiffness and swelling, reduced fatigue, and increased energy levels) are very important from parents’ perspectives. In addition to identifying crucial treatment outcomes for children and their caregivers, these qualitative findings provided valuable insights into selecting outcomes for future trials and guiding everyday clinical practice.
	Variation in outcomes^a^	Explains differences in outcomes between clusters or participants in the trial	4 (<1%)	1 (<1%)	Griffin et al. (50), within a multicenter feasibility RCT treating distal femur fracture, undertook semi-structured interviews with 11 patients or their consultees and 24 staff. The qualitative research indicated that a variety of staffing structures, research cultures, as well as patient populations and characteristics resulted in very diverse ways in which the trial was carried out at each site, which may explain why the number of patients who are eligible for enrolment but are not recruited varies from site to site.
	Results dissemination^c^	Identifying methods for disseminating trial results to a broader audience	2 (<1%)	1 (<1%)	Francis-Auton et al. (51) undertook semi-structured interviews with 5 GPs, 9 audiologists with experience of cochlear implant (CI) or hearing aids (HA), 9 HA users, and 5 HA user supporters before a proposed RCT on sensorineural hearing loss. The qualitative study identified the preferred ways of accessing trial results for audiologists and patients respectively. In addition, several local hearing loss services were identified as potential partners in the dissemination of trial results to a broader audience.
Measures of process and outcome			13 (1.7%)	8 (3.3%)	
	Accuracy of measures^a^	Assesses validity of process and outcome measures in the trial	11 (1.4%)	6 (2.5%)	Moyle et al. (52), as part of a cluster RCT investigating the efficacy of robots in accompanying people with dementia, analyzed 5 of 138 participants’ interaction videos recorded in weeks 1, 5, and 10. Qualitative findings suggested that patients’ emotions and responses to the robot could vary significantly over time, which led investigators to reflect on the bias of using a few assessment time points and consider employing frequent measurements in trials targeting people with dementia to generate comprehensive and balanced results.
	Development of outcome measures^a^	Contributes to the development of a new process and secondary outcome measures	2 (<1%)	2 (<1%)	Erridge et al. (53) undertook four semi-structured interviews and an FGD with patients (preoperative or postoperative) before a proposed double-blinded RCT relieving pain via cannabis-derived products. They suggested that nausea and vomiting are both critical outcomes for patients and there is a call for developing reliable measurements, which shall be taken multiple times per day to cope with the nature of nausea, which is fleeting and hard to scale.
Target condition			64 (8.2%)	14 (5.8%)	
	Experience of the disease, behavior, or beliefs^a^	Explores the experience of having or treating a condition that the intervention is aimed at, or a related behavior or beliefs	34 (4.4%)	10 (4.1%)	Berger et al. (54) applied several qualitative methods with 14 women from 3 arms of an open-label RCT treating primary dysmenorrhea, including semi-structured interviews, body portrayals of subjective pain experiences in different colors before and after the intervention, and open questionnaires 1 year after. The qualitative research provided in-depth insights about the dynamic fluctuations of pain intensity, the organs where different types of pain occurred (such as dull and sharp pain), changing perceptions of temperature (such as the cold of feet), and fluctuating emotions of restlessness. These insights contribute to a comprehensive understanding of dysmenorrhea in all its manifestations and the effectiveness of interventions.
	Experience of the body, behavior, or beliefs^c^	Exploring the body experiences relating to specific physiological processes or organs	4 (<1%)	0	Mukunya et al. (55) undertook in-depth interviews with 9 professionals, 10 mothers, and 3 men, plus 3 FGDs with young and older women as part of a phase IV RCT of preventing newborn infections via using chlorhexidine in cord cleansing. They noted that the cord is endowed with cultural meanings by Ugandans: not only a symbol of life (in utero) but a symbol of anxieties and family ties (after birth). The qualitative research interpreted why the mother cannot make the treatment choice regarding umbilical cord independently.
	Awareness, help seeking, or expectation^c^	Investigating help seeking behaviors, or treatment expectations during the patient journey	26 (3.4%)	4 (1.6%)	Weckesser et al. (30) used FGDs and telephone interviews with 21 selected women who had undergone a cesarean section within 6 months before a pilot RCT reducing endometritis and preventing sepsis. Qualitative research indicated that changes in wound color or dehiscence are some of the triggers that influence women’s decision to seek medical treatment.

a Original category.

b Modified category.

c Newly added category.

Drawing on all identified examples, sixty research questions that could be addressed by qualitative studies with RCTs were extrapolated (Table 2). The questions cover a wide range of topics, including designing and developing the intervention, validating its effectiveness, implementing the intervention in the real world, and understanding the preferences among different interventions for various stakeholders (e.g., healthcare providers, patients, and policymakers), as well as the key factors influencing their decision making. These questions illustrate the nuanced complexities of drugs and devices in controlled trials and the real world. For example, consider the benefits of an intervention: in addition to understanding which benefits can be perceived by stakeholders (question 5), it is also crucial to consider whether these perceived benefits are attributable to the intervention itself or to ancillary components or procedures associated with its delivery (question 6). Additionally, questions about how perceptions of benefits evolve over time (question 7) and how different people perceive benefits (question 9) highlight the temporal and individual variability in the trial context. It is equally important to recognize the knowledge gap between the objective existence of benefits and users’ perception of them, which highlights the necessity to understand the complex process by which users gradually become aware of, interpret, and internally construct the belief that their physical or mental conditions have improved as a result of the drug or device (question 8).

**Table 2.** Examples of questions that can be addressed by using qualitative research in trials Table 2. long description.

Category	Typical questions	Representative studies
Intervention content and delivery	01 What expectations, needs, or insights from stakeholders can guide the design of future interventions?	(34;56;57)
	02 How could the intervention be improved?	(33;58;59)
	03 What is the mechanism that makes the intervention effective?	(32;60;61)
	04 What factors activated or inhibited the mechanism by which the intervention produced its effects? Example: Harrison et al. (62) undertook five focus group discussions within a pilot cluster RCT on neonatal infections. Qualitative findings indicated that limited literacy and translation issues hindered the intended mechanism – communicating health messages through combined text and images – resulting in participants concentrating on the images only and overlooking the written messages, thereby preventing the alcohol-based hand rub from achieving its full potential.	(62–64)
	05 What kind of benefits do stakeholders perceive?	(39;65;66)
	06 To what extent were perceived benefits attributable to the intervention? Example: Benton et al. (67) conducted semi-structured interviews with 32 participants from an RCT comparing the effectiveness of two cardiotocography monitoring technologies. Qualitative findings suggest that women’s perceived benefits during the trial were attributed more to whether a fetal scalp electrode (FSE) had been used than to the assigned arm (Stan + CTG or CTG alone).	(26;67;68)
	07 How did perceived benefits change over time?	(49;69;70)
	08 How were the benefits of the intervention perceived, and what was required for them to be perceived? Example: Lendaro et al. (71) conducted observations before a double-blind RCT on phantom limb pain. Qualitative findings indicated that patient S4 initially recognized the value of phantom motor execution after she unexpectedly fell asleep without pain even though she was not taking hydrocodone and refined this perception through ongoing adjustments to her painkiller dosage.	(33;71;72)
	09 Do all users perceive the benefits of the intervention?	(73–75)
	10 To what extent is the intervention acceptable in principle, and for whom?	(34;38;76)
	11 To what extent was the intervention acceptable in practice, and for whom?	(32;37;77)
	12 What factors were facilitators and barriers to feasibility and acceptability of the intervention?	(33;78;79)
	13 Which stakeholders had a significant impact on feasibility and acceptability of the intervention?	(80–82)
	14 What were the changes in feasibility and acceptability over the course of the intervention?	(27;83;84)
	15 Were there any deviations from the protocol?	(50;59;60;85)
	16 How well was the intervention adhered to?	(86;87)
	17 How did adherence to the intervention change over time?	(71;72)
	18 What were the facilitators and barriers to compliance?	(64;84;88)
	19 In trials involving two or more interventions, what were the similarities and differences in the factors affecting adherence between the different interventions? Example: Hagen et al. (72) undertook longitudinal qualitative research within a multicenter RCT on urinary incontinence. They suggested that some participants experienced additional barriers to treatment adherence when using biofeedback-mediated technology compared to conventional pelvic floor muscle training, including a sense of intrusiveness, and a lack of privacy at home, etc.	(72)
	20 Do the factors that influence adherence to the intervention in everyday settings differ from those observed in clinical trials?	(72)
	21 In what ways did users integrate interventions into their daily lives?	(71;89)
	22 Who were the major stakeholders who influenced compliance?	(36;48;90)
	23 What were the facilitators and barriers to the intervention’s implementation in everyday life?	(63;91;92)
	24 What can be done to improve the implementation of the intervention?	(88;92;93)
	25 What was the difference between dominant practice in certain cultural contexts and the interventions listed in the research protocol?	(68;94)
	26 How do stakeholders understand prioritization, substitution, and/or complementarity among different interventions? Example: Watkins et al. (69) as part of a pragmatic open-label RCT on bacterial vaginosis, conducted semi-structured interviews with participants from both trial arms at 6 of the 22 sites. Qualitative findings suggested that individuals tended to manage mild symptoms with lactic acid gel, which was perceived as a gentler option that helped avoid the stigma associated with seeking antibiotic treatment at a clinic; however, when symptoms became more severe, they turned to oral metronidazole, despite concerns about potential side effects.	(26;50;95)
	27 What factors influenced the attitudes and choices of participants toward different interventions?	(47;69;70;96)
	28 To what extent did the intervention affect interpersonal relationships?	(10;39;97)
Trial design, conduct, and processes	29 What were the barriers and facilitators to trial recruitment and retention?	(29;98;99)
	30 What preexisting factors (e.g., beliefs, knowledge, and context) shaped health professionals’ attitudes toward trial participation and their performance in the role of researcher? Example: Papoutsi et al. (100) undertook interviews and observations at seven sites of an RCT on home monitoring in patients with heart failure. Qualitative findings indicated that, in some sites, clinical staff limited patient referrals or actively resisted cooperation due to several concerns (e.g., believing that the protocol conflicted with usual care), whereas in sites lacking specialist nursing resources, clinicians were more inclined to engage in trials, viewing them as an opportunity to relieve service pressures.	(50;100;101)
	31 In what ways did trainings and preparations have an impact on the trial and the stakeholders involved?	(29;42;102)
	32 What were the challenges in implementing training and/or preparation for the trial?	(28;103)
	33 What were the needs and suggestions regarding training and preparation for the trial?	(50;99;104)
	34 Is the trial acceptable in principle, and for whom?	(38;49;51)
	35 To what extent did stakeholders find this trial feasible and/or acceptable in practice?	(43;47;105)
	36 How do healthcare professionals make clinical decisions in everyday settings? Example: Crawford et al. (106), as part of a pilot RCT on patients with schizophrenia and related psychosis, conducted interviews with 51 health professionals. Clinicians believed that stabilizing patients’ mental states is far more important than discussing side effects (e.g., sexual dysfunction) before health improvement, since most patient interactions occurred during episodes of acute illness. In addition, clinicians may marginalize discussions about sexual dysfunction in everyday practice due to professional norms and certain cultural contexts.	(49;106;107)
	37 What did participants think of the post-trial care?	(45;108)
	38 Did the trial raise any ethical concerns among stakeholders?	(51;52;109)
	39 What were the participants’ views on informed consent?	(30)
	40 How did participants understand key information about the trial?	(28;47;110)
	41 In what ways did health professionals’ descriptions of treatment options affect participant or family preferences in the trial?	(29;111)
	42 How much control did participants have over their trial participation?	(29;50;112)
	43 In what ways did patients and their caregivers agree or disagree on treatment preferences? Did this influence clinical decision-making by healthcare professionals?	(29)
	44 What were the possible privacy implications of the outcome measurement process in the trial?	(36;109)
	45 What did researchers do to promote the health of participants after the trial?	(45)
	46 In what ways can the trial be adapted to be contextually and culturally sensitive with different trial areas and/or populations?	(46;109)
	47 What impacts did the trial have on participant’s and/or health professional’s everyday lives or work?	(38;102;113)
	48 How did nonintervention components affect participants’ behaviors and attitudes in the trial?	(48;114;115)
Outcomes and measures	49 Which outcome measures were most important to stakeholders?	(49;116;117)
	50 What may explain differences in results between study sites and/or study populations?	(50;75;118)
	51 In what ways were the study’s measurements affected (i.e., biased and confounded), and why? Example: Re et al. (107), as part of a pilot open label RCT on adolescent first episode psychosis, undertook semi-structured interviews with 17 clinicians. They revealed that although the trial was nominally randomized, clinicians often relied on their own judgment in practice, making individualized treatment decisions with adolescents based on their level of distress and health risks.	(52;64;107)
	52 In what ways, if at all, did the disclosures of intervention information affect the attitudes and behaviors of the participants in the trial?	(119)
	53 How could the outcome measures be improved to achieve comprehensive and reliable results?	(52;53;105)
	54 What are the best ways to communicate the trial results with different stakeholders?	(51;99)
Target condition	55 How do patients acquire, process, or comprehend information about diseases and/or health conditions and associated health technologies? Example: Eveleigh et al. (120), within a single-blinded RCT on discontinuing antidepressant use, conducted semi-structured interviews with 16 participants who had been using antidepressants long-term despite having no current corresponding indication. They suggested that certain respondents viewed antidepressants as essential supplements to replenish “missing substances” in their bodies, thereby accepting long-term drug dependence.	(64;120;121)
	56 What were the symptoms or conditions that patients experienced?	(54;69;117)
	57 In what ways do patient’s health conditions affect everyday life and relationships?	(70;115;122)
	58 To what extent do patient’s cultural beliefs about the body influence healthcare decision making?	(55;123)
	59 What do patients expect from the treatment process and/or outcome?	(46;124;125)
	60 What influences whether and when people receive treatment or further investigation? Example: Rapport et al. (70) undertook 35 semi-structured interviews with 20 participants as part of a pragmatic RCT investigating the efficacy of infliximab in treating patients with acute severe ulcerative colitis. Qualitative findings indicated that patients were often misdiagnosed in the early stages of the disease and were not referred for endoscopic examination until their symptoms worsened, particularly when weight loss occurred.	(30;33;121)

## Discussion

This review identified a significant increase in the number of qualitative studies conducted with RCTs, rising from 296 articles published over 33 months (2008–2010) (1) to 1,121 over 24 months a decade later (2019–2020). For qualitative studies specifically focusing on drug and device trials, the number of publications rose from ~80 articles (7) to 207 over the same respective periods. There are considerable similarities between the characteristics of the included studies in this review and those in O’Cathain et al.’s review (1). Around one-third of the qualitative studies that we identified were also conducted at the pretrial stage. Moreover, examples focusing on the intervention content and delivery, and on trial design and conduct, constituted the largest and second-largest categories, respectively. Nevertheless, the distribution of examples in different categorizations also implied that the functionality and potential value of qualitative research in terms of trial outcomes, measures, ethics, and stakeholder beliefs and behaviors might be under-utilized and not fully explored. Six out of the twelve potential values of using qualitative research to the generation of effectiveness evidence posited in O’Cathain et al.’s framework (1) were corroborated by identifying specific follow-up actions resulting from the qualitative findings. Our framework, revised for studies involving drugs and devices, indicates that the contribution of qualitative research in clinical trials goes beyond the trial itself.

This review identified emerging subcategories about the application of qualitative research conducted with RCTs. In comparison to previous subcategories influenced by the concept of process evaluation (3), the newer categories not only place a greater emphasis on human beings (rather than materials or data) and their subjectivity, but also highlight the importance of issues after the research process, such as the dissemination of results (126) and the acceptability of post-trial healthcare arrangements. Through the perspective of health professionals, patients, carers, and society, the newly added subcategories recognized how the complexity of human perceptions, emotions, and behaviors could influence the conduct of trials and interventions, for example, the training process and staff performance in the trial, resonating the argument of how subjective and social processes provides crucial value in ensuring scientific evidence is translated into practice (127). The subcategory “completion of outcome measures,” identified only once in the original review, did not recur in our review and was therefore not retained in our categorization. This may reflect that, in drug and device trials, issues related to the completion of outcome measures are less likely to be the focus of qualitative investigations.

The current study contributes to the understanding of the appropriate timing for qualitative research in drug or device trials. Several temporal frameworks have cited the benefits of selecting appropriate outcomes and measures as a rationale for conducting qualitative research at the pretrial stage (128). While the previous review has suggested a different conclusion (1), the current research is consistent with this assumption, as qualitative studies conducted at the pretrial stage included a higher proportion of examples in the categories of outcome (3.3 percent vs. 1.8 percent) and measures of process and outcome (3.3 percent vs. 1.7 percent) compared with all included studies. Our analysis also shows that qualitative studies aimed at guiding intervention design and development were more often conducted alongside large-scale trials. This is different from what a previous review found (1), which primarily focused on complex interventions and found that such work was more commonly conducted during the pretrial stage. This difference may reflect the distinct research and development pathways of drugs and devices, which tend to prioritize safety and efficacy over contextual adaptation and subjective experience in the early phases.

There has been considerable discussion regarding the importance and challenges of reporting on the impact of qualitative research on trials (4;129). Despite this, most of the included studies did not explicitly articulate how the application of qualitative research had changed or influenced specific trials and/or interventions, such as improving external validity, and so forth One explanation is that some investigations (especially those without an embedded qualitative component) failed to integrate qualitative and quantitative data (e.g., whether qualitative findings would be used to explain effectiveness results depended on research timeline and available funding support) (130). The reasons for such omissions may be related to time constraints, perceptions of the credibility of the qualitative evidence, or resistance to changing established trial practice or ways of thinking (3). Another barrier is the concern that discussing qualitative findings within the research team poses potential risks associated with the conduct of the trial (e.g., disclosing the limited acceptability of the intervention among stakeholders might affect staff motivation in patient recruitment) (130). It has been argued that these risks can be mitigated by establishing a committee for reviewing process evaluation data and addressing significant ethical and acceptability concerns before they become critical (131). In addition to factors embedded during trial design and conduct, it is essential to indicate the challenges in inferring the causal relationships between some qualitative insights and subsequent outcomes (e.g., an increase in recruitment rates) (29). Moreover, values that necessitate prolonged observation and deliberate evaluation to be accurately perceived or measured, such as enhancing the transferability of results to real-world settings, may be challenging to recognize in the short term, or at least before the implementation of full-scale interventions. Rather than stating that this study quantitatively confirms half of the potential values proposed in the earlier framework (1), we prefer to emphasize that our findings corroborate those values that are less constrained by measurement limitations.

Existing research (132;133) indicates that qualitative studies conducted with trials appear less frequently in high-impact medical journals, which may reflect ongoing challenges to the visibility of qualitative research. Further evidence of the secondary status of qualitative research is the fact that included studies seldom explicitly state the start and end dates for the collection and analysis of qualitative data (1;3). There was evidence that some qualitative studies were conducted long after trials had started (134), while some were completed much earlier than the trials (130), which may prevent qualitative research from delivering its full value. Perhaps a more important question is how the timing of qualitative research in clinical studies is determined and by whom (135).

While a similar number of qualitative studies in this review were conducted in high-income countries as well as low- and middle-income countries, the latter are underrepresented compared to their significantly greater share of the global population (6). The structural marginalization of participants’ nuanced needs and preferences may be costly, particularly when these are shaped by differences in geography, economics, social, and cultural contexts that often reflect real-world conditions in which drugs and devices would ultimately be implemented. It can hinder researchers from fully comprehending the uncertainties associated with complex interventions and evaluations, as well as prevent them from developing and refining sustainable and contextually flexible intervention components before scaling up the intervention (136).

### Strengths and limitations

A key strength of this study is that it relied on a large number of published qualitative studies, which were conducted alongside actual RCTs involving drugs and medical devices. This focus makes our findings highly applicable to the design, evaluation, and implementation of most life science interventions and provides field-specific categorizations of the value of using qualitative research. Additionally, a list of representative questions was developed to illustrate how qualitative methods could be employed to address diverse research aims under various circumstances. This could prove a useful resource for a variety of audiences (e.g., trial investigators and sponsors, journal editors and peer reviewers, research ethics reviewers, and funders), especially those less familiar with collecting and analyzing nonnumerical data.

While an existing framework guided the categorization of examples, we acknowledge that our subjectivity might have influenced how the examples were interpreted. Although efforts were made to align examples with appropriate subcategories, we recognize that other researchers might have categorized the selected examples differently based on their perspectives or experiences. Besides, a lack of clarity in reporting when qualitative studies were conducted alongside RCTs may have limited a more nuanced understanding of the context in which these values were generated. We are also aware that the exclusion of gray literature and non-English publications may limit the global representativeness and completeness of the findings. This study represents a methodological snapshot of recent research practices and may not fully capture how qualitative research is conducted alongside trials involving more recently emerging technologies, given its temporal coverage from 2019 to 2020. Although the overall categorization of the values of qualitative research is less likely to have changed substantially, the relative frequency of certain subcategories within this framework may vary across time.

## Conclusions

The number of qualitative studies conducted with RCTs has significantly increased compared to a decade ago, and trials involving drugs and devices are no exception. Although qualitative research has been more commonly used to optimize trial design and conduct, as well as to improve the development and implementation of interventions, its considerable potential in understanding outcomes, enhancing the validity of measures, and understanding experiences and behaviors related to conditions continues to be underutilized. Furthermore, strengthening qualitative research in geographic regions and therapeutic areas with limited qualitative inquiry may enhance the contextual relevance and impact of trials and their associated interventions. While qualitative methods have traditionally been associated with complex interventions, this review – focusing on trials of drugs and medical devices – contributes to expanding the relevance of using qualitative research with trials across a broader range of intervention types. The illustrative list of questions proposed by this mapping review can assist investigators, intervention developers, and regulators in identifying where and how qualitative methods can be meaningfully applied at different stages of the trial process, thereby maximizing opportunities for the value they might bring.

## Supporting information

10.1017/S0266462326103936.sm001 Huang et al. supplementary material Huang et al. supplementary material

## Data Availability

All data relevant to this study are included in the article or uploaded as Supplementary Material.

## Supplementary material

The supplementary material for this article can be found at http://doi.org/10.1017/S0266462326103936.

## Long descriptions

### Figure 1. Long description

The flowchart is organized into three vertical phases labeled on the left: Identification, Screening, and Included.

1. Identification Phase:

- Top-left box: Records identified from (n = 9,574) including CINAHL (n = 2,643), Embase (n = 2,484), MEDLINE (n = 2,466), ASSIA (n = 932), PsycINFO (n = 926), and NIHR Journals Library (n = 123).

- Top-right box (exclusion): Records removed before screening: Records marked as duplicate by automation tools (n = 2,467) and Records marked as duplicate by a human (n = 12).

2. Screening Phase:

- Second box down: Records screened (n = 7,095). An arrow points right to Records excluded (n = 5,346).

- Third box down: Reports sought for retrieval (n = 1,749). An arrow points right to Reports not retrieved (n = 5).

- Fourth box down: Reports assessed for eligibility (n = 1,744). An arrow points right to Reports excluded: Not qualitative research (n = 291), Not related to a specific RCT (n = 211), Not a journal article (n = 90), Not reporting empirical findings (n = 27), and Other (n = 4).

- Fifth box down: Reports eligible (n = 1,121). An arrow points right to Reports excluded: Not related to an RCT involving drugs or medical devices (n = 914).

3. Included Phase:

- Final bottom box: Studies included in review (n = 207).

Navigate back to Figure 1..

### Figure 2. Long description

The diagram consists of two connected flow charts.

Left Panel: Health technologies to Therapeutic areas.

The left axis lists four categories: Medicines 83, Conventional devices 67, Digital devices 66, and Vaccines 3.

Flow lines connect these to the central axis of Therapeutic areas, which includes:

* Certain infectious and parasitic diseases 47

* Other 31

* Mental and behavioural disorders 26

* Diseases of the circulatory system 16

* Diseases of the respiratory system 16

* Diseases of the nervous system 14

* Pregnancy, childbirth and the puerperium 12

* Endocrine, nutritional and metabolic diseases 12

* Neoplasms 11

* Diseases of the genitourinary system 9

* Diseases of the digestive system 9

* Diseases of the musculoskeletal system and connective tissue 8

* Injury, poisoning and certain other consequences of external causes 8

Right Panel: Therapeutic areas to Qualitative research locations.

The central axis of Therapeutic areas flows into the rightmost axis of Qualitative research locations:

* United Kingdom 80

* North America 38

* Africa 37

* Europe Ex UK 32

* Oceania 22

* Asia 10

Wider flow lines represent a larger number of studies. The United Kingdom is the most frequently represented location for qualitative research overall.

Navigate back to Figure 2..

### Table 1. Long description

The table is organized into six columns: Category, Subcategory, Description, Frequency in 776 examples (%), Frequency in 243 pre-trial examples (%), and Example.Frequencies below are reported first for all 776 examples and then for the 243 pre-trial examples.

1. Intervention content and delivery: Total frequency 493 (63.5%); pre-trial frequency 150 (61.7%). Subcategories include:

* Intervention development and adjustment: 39 (5.0%); pre-trial 14 (5.8%).

* Models, mechanisms, and underlying theory development: 25 (3.2%); pre-trial 7 (2.9%).

* Perceived value and benefits of intervention: 109 (14.0%); pre-trial 32 (13.2%).

* Acceptability of intervention in principle: 5 (<1%); pre-trial 4 (1.6%).

* Feasibility and acceptability of intervention in practice: 151 (19.5%); pre-trial 50 (20.6%).

* Compliance with intervention: 43 (5.5%); pre-trial 12 (4.9%).

* Implementation of the intervention in the real world: 64 (8.2%); pre-trial 16 (6.6%).

* Perceptions and treatment choices between different interventions: 43 (5.5%); pre-trial 13 (5.3%).

* Private life influenced by the intervention: 14 (1.8%); pre-trial 2 (<1%).

2. Trial design, conduct, and processes: Total frequency 192 (24.7%); pre-trial frequency 63 (25.9%). Subcategories include:

* Recruitment and retention: 48 (6.2%); pre-trial 15 (6.2%).

* Engagement and performance of health professionals in trial: 24 (3.1%); pre-trial 7 (2.9%).

* Preparation and training: 19 (2.4%); pre-trial 5 (2.1%).

* Feasibility and acceptability of the trial in principle: 5 (<1%); pre-trial 5 (2.1%).

* Feasibility and acceptability of the trial in practice: 48 (6.2%); pre-trial 19 (7.8%).

* Acceptability of post-research care arrangements: 4 (<1%); pre-trial 1 (<1%).

* Ethical conduct: 20 (2.6%); pre-trial 6 (2.5%).

* Adaptation of trial conduct to local context: 2 (<1%); pre-trial 0.

* Impact of trial on staff, researchers, or participants: 17 (2.2%); pre-trial 4 (1.6%).

* Role of trial components other than interventions: 5 (<1%); pre-trial 1 (<1%).

3. Outcomes: Total frequency 14 (1.8%); pre-trial frequency 8 (3.3%). Subcategories include:

* Breadth of outcomes: 8 (1.0%); pre-trial 6 (2.5%).

* Variation in outcomes: 4 (<1%); pre-trial 1 (<1%).

* Results dissemination: 2 (<1%); pre-trial 1 (<1%).

4. Measures of process and outcome: Total frequency 13 (1.7%); pre-trial frequency 8 (3.3%). Subcategories include:

* Accuracy of measures: 11 (1.4%); pre-trial 6 (2.5%).

* Development of outcome measures: 2 (<1%); pre-trial 2 (<1%).

5. Target condition: Total frequency 64 (8.2%); pre-trial frequency 14 (5.8%). Subcategories include:

* Experience of the disease, behavior, or beliefs: 34 (4.4%); pre-trial 10 (4.1%).

* Experience of the body, behavior, or beliefs: 4 (<1%); pre-trial 0.

* Awareness, help seeking, or expectation: 26 (3.4%); pre-trial 4 (1.6%).

Navigate back to Table 1..

### Table 2. Long description

The table is organized into three columns: Category, Typical questions, and Representative studies.

1. Intervention content and delivery (Questions 01 to 28): Focuses on stakeholder expectations, mechanisms of effectiveness, perceived benefits, acceptability, feasibility, adherence, and implementation in everyday life. Notable examples include Harrison et al. on neonatal infections and Lendaro et al. on phantom limb pain.

2. Trial design, conduct, and processes (Questions 29 to 48): Addresses barriers to recruitment, health professionals’ attitudes, training needs, ethical concerns, informed consent, and cultural sensitivity. An example includes Papoutsi et al. on home monitoring for heart failure patients.

3. Outcomes and measures (Questions 49 to 54): Explores which measures are important to stakeholders, differences between study sites, potential biases, and communication of results. Re et al. is cited regarding clinician judgment in adolescent psychosis trials.

4. Target condition (Questions 55 to 60): Examines how patients comprehend health conditions, the impact of symptoms on daily life, cultural beliefs, and factors influencing when people seek treatment. Examples include Eveleigh et al. on antidepressant use and Rapport et al. on ulcerative colitis.

Navigate back to Table 2..
